# *HOXB13:IL17BR *and molecular grade index and risk of breast cancer death among patients with lymph node-negative invasive disease

**DOI:** 10.1186/bcr3402

**Published:** 2013-03-14

**Authors:** Laurel A Habel, Lori C Sakoda, Ninah Achacoso, Xiao-Jun Ma, Mark G Erlander, Dennis C Sgroi, Louis Fehrenbacher, Deborah Greenberg, Charles P Quesenberry

**Affiliations:** 1 Division of Research, Kaiser Permanente, 2000 Broadway, Oakland, CA 94612, USA; 2 bioTheranostics, 9640 Towne Centre Dr. Suite 200, San Diego, CA 92121, USA; 3 Molecular Pathology Unit, Massachusetts General Hospital,, 149 13th Street, Charlestown, MA 02129, USA; 4 Hematology/Oncology Department, Kaiser Permanente Medical Center, 975 Sereno Drive, Vallejo, CA 94589, USA; 5 TPMG Regional Laboratory, Kaiser Permanente, 1725 Eastshore Highway, Berkeley, CA 94710, USA

## Abstract

**Introduction:**

Studies have shown that a two-gene ratio (*HOXB13:IL17BR*) and a five-gene (*BUB1B, CENPA, NEK2, RACGAP1, RRM2*) molecular grade index (MGI) are predictive of clinical outcomes among early-stage breast cancer patients. In an independent population of lymph node-negative breast cancer patients from a community hospital setting, we evaluated the performance of two risk classifiers that have been derived from these gene signatures combined, MGI+*HOXB13:IL17BR *and the Breast Cancer Index (BCI).

**Methods:**

A case-control study was conducted among 4,964 Kaiser Permanente patients diagnosed with node-negative invasive breast cancer from 1985 to 1994 who did not receive adjuvant chemotherapy. For 191 cases (breast cancer deaths) and 417 matched controls, archived tumor tissues were available and analyzed for expression levels of the seven genes of interest and four normalization genes by RT-PCR. Logistic regression methods were used to estimate the relative risk (RR) and 10-year absolute risk of breast cancer death associated with prespecified risk categories for MGI+*HOXB13:IL17BR *and BCI.

**Results:**

Both MGI+*HOXB13:IL17BR *and BCI classified over half of all ER-positive patients as low risk. The 10-year absolute risks of breast cancer death for ER-positive, tamoxifen-treated patients classified in the low-, intermediate-, and high-risk groups were 3.7% (95% confidence interval (CI) 1.9% to 5.4%), 5.9% (95% CI 3.0% to 8.6%), and 12.9% (95% CI 7.9% to 17.6%) by MGI+*HOXB13:IL17BR *and 3.5% (95% CI 1.9% to 5.1%), 7.0% (95% CI 3.8% to 10.1%), and 12.9% (95% CI 7.1% to 18.3%) by BCI. Those for ER-positive, tamoxifen-untreated patients were 5.7% (95% CI 4.0% to 7.4%), 13.8% (95% CI 8.4% to 18.9%), and 15.2% (95% CI 9.4% to 20.5%) by MGI+*HOXB13:IL17BR *and 5.1% (95% CI 3.6% to 6.6%), 18.6% (95% CI 10.8% to 25.7%), and 17.5% (95% CI 11.1% to 23.5%) by BCI. After adjusting for tumor size and grade, the RRs of breast cancer death comparing high- versus low-risk categories of both classifiers remained elevated but were attenuated for tamoxifen-treated and tamoxifen-untreated patients.

**Conclusion:**

Among ER-positive, lymph node-negative patients not treated with adjuvant chemotherapy, MGI+*HOXB13:IL17BR *and BCI were associated with risk of breast cancer death. Both risk classifiers appeared to provide risk information beyond standard prognostic factors.

## Introduction

Previously, it was shown that a simple homeobox B13:interleukin 17 receptor B two-gene ratio (hereafter referred to as *HOXB13:IL17BR*) could predict recurrence in a sample of patients with estrogen receptor (ER)-positive breast cancer receiving adjuvant tamoxifen therapy [1]. Subsequent results suggest that *HOXB13:IL17BR *may be both prognostic (that is, predictive of disease outcome) and predictive of tamoxifen benefit (that is, tamoxifen response/resistance) [2-4].

More recently, a five-gene (budding uninhibited by benzimidazoles 1 homolog beta (*BUB1B*), centromere protein A (*CENPA*), never in mitosis gene a-related kinase 2 (*NEK2*), Rac GTPase-activating protein 1 (*RACGAP1*), ribonucleotide reductase M2 (*RRM2*)) tumor grade signature (MGI for molecular grade index) was developed to recapitulate tumor grade. In one study, MGI predicted clinical outcome of early-stage breast cancer patients with comparable performance to much more complex gene signatures [5]. Furthermore, MGI and *HOXB13:IL17BR *have been used together (hereafter referred to as MGI+*HOXB13:IL17BR*) to stratify ER-positive lymph node-negative patients treated with endocrine therapy into three risk groups (low, intermediate, and high) [5]. Both signatures have also been newly combined to derive a patient risk score (range: 0 to 10), reflective of the rate of distant metastasis at 10 years post-diagnosis, known as the Breast Cancer Index (BCI) [6].

The purpose of this study was to evaluate the performance of MGI+*HOXB13:IL17BR *and BCI, as defined previously [5,6], in an independent study population of ER-positive, lymph node-negative breast cancer patients who were not treated with chemotherapy. A prespecified primary aim was to assess the degree to which the MGI+*HOXB13:IL17BR *risk classifier predicts the risk of breast cancer-specific mortality among tamoxifen-treated ER-positive, node-negative patients, either alone or after accounting for tumor size and tumor grade. A prespecified secondary aim was to similarly examine the extent to which the MGI+*HOXB13:IL17BR *predicts the risk of breast cancer-specific mortality among tamoxifen-untreated ER-positive, node-negative patients. With the recent development of the BCI, the study aims were expanded to include a parallel evaluation of this newer risk classifier.

## Materials and methods

### Study population and design

We conducted a case-control study nested within a cohort of 4,964 potentially eligible breast cancer patients. This same patient population was used in a previously described study of Oncotype DX (Genomic Health, Inc., Redwood City, CA, USA) [7]. The study was approved by the Kaiser Permanente Northern California Institutional Review Board (IRB). Informed consent from study participants was waived by the IRB.

Briefly, the Northern California Kaiser Permanente tumor registry was used to identify all female health plan members aged less than 75 years who were diagnosed with lymph node-negative invasive breast cancer from 1985 to 1994. Breast cancer patients were eligible if their initial disease was not treated with chemotherapy. Patients were excluded for the following reasons: inflammatory or bilateral breast cancer or evidence of metastasis (including lymph nodes) at initial diagnosis; prior invasive cancer (breast or other) at diagnosis; or unknown/unconfirmed treatment with tamoxifen.

Patients were followed until death due to breast cancer, death from another cause, bilateral breast cancer, termination of membership, or December 2002, whichever came first. Cases were patients whose first event was death from breast cancer. For each case, up to three controls were randomly selected from patients alive and under follow-up at the time of the case's death (that is, risk set sampling) [8]. Cases and controls were matched on age (within one year), race (non-Hispanic white, Hispanic, Black, Asian), calendar year of diagnosis (exact year), Kaiser Permanente pathology department of origin, and treatment of index breast cancer with tamoxifen (yes, no). Initial diagnosis, treatment, and cause of death were confirmed through medical chart review.

Of the 402 cases identified as potentially eligible by the tumor registry, 269 were determined to be eligible by chart review. Of the 989 controls initially matched to the identified cases, 722 were determined to be eligible by chart review. Of those eligible by chart review, 31 cases and 91 controls were excluded because of missing tumor blocks. Four additional cases who could not be matched to at least one control were excluded, leaving 234 cases and 631 controls available for pathology-based studies.

### Blinding and batching of pathology and laboratory procedures

All pathology and laboratory procedures, including slide review, sectioning of tumor blocks, macrodissection, RT-PCR assays, and gene expression analyses, were conducted blinded to the case-control status of patient specimens. In addition, both cases and controls were included in every batch of pathology materials sent to bioTheranostics (San Diego, CA, USA) for gene expression analyses.

### Sample preparation

Breast cancer samples (three to six 10 micron unstained tissue sections plus one H&E-stained slide per sample) were provided to bioTheranostics for gene expression analysis. The H&E slides were reviewed by a pathologist at bioTheranostics to identify prominent tumor areas. Samples containing no tumor or primarily noninvasive lesions were excluded from further analysis. Of the 220 cases and 570 controls included in our previous study on Oncotype DX, there were 13 cases or controls with insufficient tumor for this study; an additional two controls became uninformative because their matched case had no tumor tissue. For the remaining samples, macrodissection by scalpel was performed on adjacent unstained sections to enrich for tumor cells. Macrodissected tissue sections were treated with 2 mg/ml Proteinase K (Invitrogen, Carlsbad, CA, USA) overnight at 50°C. Total RNA was extracted using the RNA Cleanup kit (Zymo Research, Orange, CA, USA) and treated with RNase-free DNase (Epicentre, Madison, WI, USA), and absence of genomic DNA contamination was confirmed for all samples. Adequate RNA was obtained for 191 cases and 417 controls. The distribution of factors available from the tumor registry (diagnosis year, age, race, tumor size, ER status and tamoxifen treatment) was fairly similar in the 269 chart-eligible cases compared to the 191 RT-PCR-evaluable cases. This also was true for chart-eligible and evaluable controls. However, lost cases and controls were slightly more likely to be younger or to have smaller tumors. Furthermore, the distribution of characteristics, including tumor size and grade, in this study population presented in Table 1 is quite similar to the distribution among the slightly larger group of patients in our. Oncotype DX study.

**Table 1:** Selected characteristics of the study population: 191 cases, 417 controls.

	Cases	Controls
Characteristic	n(%)	n(%)
*Matched variables*		
Age at diagnosis (years)		
<40	15(8%)	20(5%)
40-49	33(17%)	94(22%)
50-59	59(31%)	113(27%)
60-74	84(44%)	190(46%)
Race/ethnicity		
White, non-Hispanic	146(76%)	326(78%)
White, Hispanic	7(4%)	11(3%)
Black	19(10%)	42(10%)
Asian	19(10%)	38(9%)
Surgery year		
1985-1989	127(66%)	278(67%)
1990-1994	64(34%)	139(33%)
Adjuvant tamoxifen		
No	134(70%)	281(67%)
Yes	57(30%)	136(33%)
*Unmatched variables*		
Tumor size (cm)		
≤ 1.0	40(21%)	128(31%)
1.1-2.0	84(44%)	187(45%)
2.1-4.0	63(33%)	96(23%)
>4.0	4(2%)	6(1%)
Tumor grade (differentiation)^1^		
Well	21(11%)	123(29%)
Moderate	79(41%)	182(44%)
Poor	91(48%)	112(27%)
ER status from RT-PCR^2^		
Positive	145(76%)	370(89%)
Negative	46(24%)	47(11%)
PR status from RT-PCR^2^		
Positive	117(61%)	324(78%)
Negative	74(39%)	93(22%)
HER2 status from RT-PCR^2^		
Positive	33(17%)	41(10%)
Negative	158(83%)	376(90%)
*HOXB13:IL17BR*		
Low risk	71(37%)	222(53%)
High risk	120(63%)	195(47%)
MGI		
Low risk	51(27%)	197(47%)
High risk	140(73%)	220(53%)
MGI+*HOXB13:IL17BR*		
Low risk	51(27%)	197(47%)
Intermediate risk	50(26%)	108(26%)
High risk	90(47%)	112(27%)
BCI		
Low risk	55(29%)	222(53%)
Intermediate risk	54(28%)	85(20%)
High risk	82(43%)	110(26%)

^1^Modified Bloom-Richardson grading criteria; ^2^performed by Genomic Health, Inc. (ER cut points ≤ 6.5 and >6.5 units; PR cut points <5.5 and ≥5.5 units; HER2 cut points <11.5 and ≥11.5 units).

### Gene selection for *HOXB13:IL17BR *and MGI

*HOXB13 *and *IL17BR *were previously identified from a microarray analysis of 60 patients with hormone receptor-positive breast cancer treated with the standard five years of adjuvant tamoxifen therapy [1]. A simple *HOXB13:IL17BR *two-gene ratio was proposed as a novel biomarker of prognosis and response to tamoxifen.

From a microarray analysis of 36 breast cancer specimens, a set of 39 genes was identified with increased expression in high-grade tumors [5]. The high correlation with tumor grade was confirmed in a large publicly available microarray data set (Uppsala cohort). The list of 39 genes was then narrowed to five, based on functional annotation of the genes, association with clinical outcome in the Uppsala cohort, and correlation with tumor grade in an independent sample of 60 patients as described previously [5].

### Gene expression analysis by RT-PCR

Expression of 11 genes of interest (ER, progesterone receptor (PR), human epidermal growth factor receptor 2 (HER2), *HOXB13, IL17BR*, choline dehydrogenase (CHDH), *BUB1B, CENPA, NEK2, RACGAP1, RRM2*) and four normalization genes (beta-actin (*ACTB*), hydromethylbilane synthase (*HMBS*), succinate dehydrogenase complex, subunit A (*SDHA*) and ubiquitin C (*UBC*)) were measured by TaqMan RT-PCR. RNA samples were converted to cDNA by reverse transcription with SuperScript™ III (Invitrogen). Each cDNA sample was analyzed in duplicate on a 384-well plate for each gene using real-time TaqMan™ assay on an ABI 7900HT instrument (Applied Biosystems, Foster City, CA, USA). All primers and probes have been described before [2]. We used the average threshold cycle (Ct) value (ref_mean) of the four reference genes as a measure of RNA input in the RT-PCR assay. A prespecified acceptance criterion used in this study was ref_mean <29.

### Calculation of gene expression indices

All methods for processing the raw RT-PCR data and calculating *HOXB13:IL17BR *and MGI were identical to prior studies [2,5,6]. *HOXB13:IL17BR *was dichotomized at the value of 0.06 (< = 0.06 vs. >0.06) and MGI was dichotomized at the value of 0 (< = 0 vs. >0) into low- and high-risk groups. Both indices were also combined following two previously described approaches [5,6]. One approach uses *HOXB13:IL17BR *and MGI in their dichotomous form to stratify patients into three groups: low risk (low MGI), intermediate risk (high MGI but low *HOXB13:IL17BR*), and high risk (high MGI and high *HOXB13:IL17BR*). The other employs both indices in their continuous form to derive a continuous risk score (that is, BCI). The BCI is computed as a polynomial function of *HOXB13:IL17BR *(*hi*) and MGI (*mgi*) values,

$$f\left(hi,mgi\right)=0.4431*mgi+0.4972*hi-0.09*hi^{3},$$

and scaled to range from 0 to 10 as follows:

$$BCI=\left\{\begin{array}{cc} 0 & \text{if}\;f\left(hi,mgi\right)<-2.5\\ \left(2*f\left(hi,mgi\right)\right)+5 & \text{if}\;-2.5\;\leq \;f\left(hi,mgi\right)\leq \;2.5\\ 10 & \text{if}\;f\left(hi,mgi\right)>2.5 \end{array}\right)$$

It has been also used to stratify patients into three risk groups: low (BCI <5), intermediate (5 ≤ BCI <6.4), and high (BCI ≥ 6.4).

### Quality control assessment

The potential impact of within-person heterogeneity and batch heterogeneity was examined. Each batch of samples to bioTheranostics included two blinded internal controls. These controls came from seven different patients, each of which was in multiple (four to eight) batches of samples (total of 17 batches). Of 34 internal controls sent, only 23 had sufficient RNA from five patients, which were included in 16 of the 17 batches. With respect to risk classification by MGI, all replicate controls were concordant for each patient. With respect to risk classification by *HOX13:IL17BR *and MGI+*HOX13:IL17BR*, all replicates for four patients and two of three replicates for one patient were concordant. With respect to risk classification by BCI, all replicates for two patients were concordant. For the remaining three patients, four of eight, two of three, and three of five replicates were concordant.

### Tumor size, grade and ER status

Tumor size was ascertained from pathology review of all H&E slides collected from all surgeries at diagnosis, when not documented in the pathology report (7% of reports). Tumor grade was assessed by two board-certified assistant professors in pathology using the modified Bloom-Richardson grading criteria [9], based on their independent reviews of an H&E slide from the most representative tumor block.

For a sizable proportion (16%) of patients, ER status of the index tumor was not indicated in the 10- to 20-year-old medical records. All tumors were therefore classified as ER-positive or ER-negative according to ER expression determined through RT-PCR by Genomic Health, Inc. (ER-positive >6.5), an approach consistent with our prior research [7]. ER status was also determined through RT-PCR by bioTheranostics (ER-positive >-2.5) [2] and through immunohistochemistry (IHC) by PhenoPath Laboratories (Seattle, WA, USA) (ER positivity >1% antigen staining). Agreement for ER status classification between Genomic Health RT-PCR and IHC was better (κ = 0.85; 95% CI: 0.78 to 0.91) [10] than that between Genomic Health and bioTheranostics RT-PCR methods (κ = 0.62; 95% CI: 0.55 to 0.70).

### Statistical analysis

Statistical analyses were conducted entirely by Kaiser Permanente researchers according to a prespecified plan, with results generated for patient groups based on ER status and tamoxifen treatment.

Conditional logistic regression was used to calculate odds ratios as estimates of the relative risks (RR) of breast cancer death associated with each risk classifier, unadjusted (univariate analyses) and adjusted for tumor size and tumor grade (multivariable analyses). Tumor size was examined both continuously in 2 cm units (for consistency with results from our prior study [7]) and categorically (≤1.0 cm, 1.1 to 2.0 cm, 2.1 to 4.0 cm, and >4.0 cm). Tumor grade was examined as a categorical variable (well differentiated, moderately differentiated, and poorly differentiated) using data from the standardized rereview. We examined tumor grade separately for pathologist 1 and pathologist 2 but present data for pathologist 1 only (for consistency with results from our prior study [7]). We also created tumor group categories that combined tumor size and grade largely using consensus group recommendations on adjuvant treatment [11,12]. The three categories were as follows: Tumor Group 1 (≤ 2.0 cm and well or ≤ 1.0 cm and moderate); Tumor Group 2 (>2.0 cm and well, or 1.1 to 2.0 cm and moderate, or ≤ 2.0 cm and poor); Tumor Group 3 (>2.0 cm and moderate/poor). Model parameters were estimated by maximum likelihood, and 95% confidence limits were calculated by the Wald method. Statistical significance was evaluated using the likelihood ratio test [13].

We performed separate analyses for women with ER-positive tumors who received or did not receive tamoxifen. Because ER status was not a matching factor but was associated with breast cancer death, only 14 of the 46 ER-negative cases were matched to ER-negative controls. Therefore, for women with ER-negative tumors who did not receive tamoxifen, we derived RR estimates from conditional logistic regression analyses of all untreated patients using terms for interaction with ER status. In addition, we derived RR estimates for each prespecified patient group from conditional logistic regression analyses of all patients using interaction terms for ER status and tamoxifen therapy. Since these results were not materially different, we present only results from analyses conducted within patient groups. Also, since the limited number of ER-negative patients yielded very imprecise estimates, we present results for ER-positive patients only.

As described previously (see Appendix in Habel *et al. *[7]), methods developed by Langholz and Borgan [14] for nested case-control data were adapted to our control sampling scheme and applied to estimate the absolute risk of breast cancer death at 10 years and corresponding 95% confidence intervals. Estimates were ascertained for prespecified categories of MGI+*HOXB13:IL17BR*, BCI, tumor size, and tumor grade for subgroups of ER-positive patients stratified on tamoxifen treatment. Ten-year risks of breast cancer death were also calculated for both treatment subgroups of ER-positive patients when cross-classified by tumor size and grade; by MGI+*HOXB13:IL17BR*, tumor size, and grade; and by BCI, tumor size, and grade.

## Results

### Characteristics of cases and controls

Breast cancer deaths occurred a median of 4.7 years after diagnosis. Among the cases and controls, the median tumor size was 1.5 cm (range 0.2 to 7.0 cm). Cases and controls were comparable with respect to matching factors including age, race, diagnosis year, and tamoxifen treatment (Table 1). Overall, about one-third of patients received adjuvant tamoxifen treatment. Prior to 1989, 10% of patients were treated with tamoxifen, whereas from 1989 to 1994, 61% of patients were treated with tamoxifen. Among those treated with tamoxifen, the median duration was four years; 10% had a year of treatment or less. Compared to controls, cases more commonly had tumors that were ER negative, PR negative, larger, or more poorly differentiated.

Cases were also more likely than controls to have tumors with higher MGI+*HOXB13:IL17BR *or higher BCI values. However, 71 (37%) of the 191 cases were discordant by the two risk classifiers. We found that 90 (47%) of 191 total cases were classified as high risk by MGI+*HOXB13:IL17BR *and 82 (43%) were classified as high risk by BCI. We also found that 111 (27%) of 417 controls were discordant by the two risk classifiers. Among the controls, we found that 197 (47%) were classified as low risk by MGI+*HOXB13:IL17BR*; 222 (53%) were classified as low risk by BCI.

For prespecified analyses stratified by ER status and tamoxifen therapy, there were 49 cases and 112 matched controls who were tamoxifen-treated and had ER-positive tumors by the RT-PCR assay. There were 90 cases and 174 matched controls who had ER-positive tumors by the RT-PCR assay and were not treated with tamoxifen. There were 12 cases and 15 matched controls who had ER-negative tumors by the RT-PCR assay and were not treated with tamoxifen. In addition, a small number of ER-negative patients were treated with tamoxifen (two cases and two matched controls).

### Distribution of risk classifier categories by tumor characteristics

All four risk classifiers were correlated with tumor size and, as expected for MGI, even more strongly with tumor grade, along with ER, PR, and HER2 status (Table 2). Women with breast tumors that were larger in size, more poorly differentiated, ER negative, PR negative, and HER2 positive were more likely to be classified as high risk. A number of patients with these same tumor characteristics, however, were classified as low risk.

**Table 2:** Distributions of tumor characteristics for categories of *HOXB13:IL17BR*, MGI, and MGI+*HOXB13:IL17BR*, and BCI.

	*HOXB13:IL17BR*		MGI		MGI+*HOXB13:IL17BR*			BCI			
		
	Low (*n *= 293)	High (*n *= 315)	Low (*n *= 248)	High (*n *= 360)	Low (*n *= 248)	Intermediate (*n *= 158)	High (*n *= 202)	Low (*n *= 277)	Intermediate (*n *= 139)	High (*n *= 192)	Total (*n *= 608)
Tumor size (cm)											
≤ 1.0	97 (58%)	71 (42%)	91 (54%)	77 (46%)	91 (54%)	39 (23%)	38 (23%)	96 (57%)	37 (22%)	35 (21%)	168
1.1 - 2.0	130 (48%)	141 (52%)	105 (39%)	166 (61%)	105 (39%)	77 (28%)	89 (33%)	119 (44%)	62 (23%)	90 (33%)	271
>2.0	66 (39%)	103 (61%)	52 (31%)	117 (69%)	52 (31%)	42 (25%)	75 (44%)	62 (37%)	40 (24%)	67 (40%)	169
Tumor grade^1^											
Well	95 (66%)	49 (34%)	103 (72%)	41 (28%)	103 (72%)	24 (17%)	17 (12%)	106 (74%)	29 (20%)	9 (6%)	144
Moderate	133 (51%)	128 (49%)	134 (51%)	127 (49%)	134 (51%)	73 (28%)	54 (21%)	144 (55%)	68 (26%)	49 (19%)	261
Poor	65 (32%)	138 (68%)	11 (5%)	192 (95%)	11 (5%)	61 (30%)	131 (65%)	27 (13%)	42 (21%)	134 (66%)	203
Tumor size and grade^2^											
Group 1	129 (66%)	66 (34%)	129 (66%)	66 (34%)	129 (66%)	42 (22%)	24 (12%)	134 (69%)	45 (23%)	16 (8%)	195
Group 2	106 (40%)	156 (60%)	80 (31%)	182 (69%)	80 (31%)	76 (29%)	106 (40%)	93 (36%)	60 (23%)	109 (42%)	262
Group 3	58 (38%)	93 (62%)	39 (26%)	112 (74%)	39 (26%)	40 (26%)	72 (48%)	50 (33%)	34 (23%)	67 (44%)	151
ER status											
Positive	274 (53%)	241 (47%)	237 (46%)	278 (54%)	237 (46%)	140 (27%)	138 (27%)	259 (50%)	119 (23%)	137 (27%)	515
Negative	19 (20%)	74 (80%)	11 (12%)	82 (88%)	11 (12%)	18 (19%)	64 (69%)	18 (19%)	20 (22%)	55 (59%)	93
PR status											
Positive	257 (58%)	184 (42%)	211 (48%)	230 (52%)	211 (48%)	126 (29%)	104 (24%)	239 (54%)	99 (22%)	103 (23%)	441
Negative	36 (22%)	131 (78%)	37 (22%)	130 (78%)	37 (22%)	23 (19%)	98 (59%)	38 (23%)	40 (24%)	89 (53%)	167
HER2 status											
Positive	12 (16%)	62 (84%)	16 (22%)	58 (78%)	16 (22%)	11 (15%)	47 (64%)	23 (31%)	16 (22%)	35 (47%)	74
Negative	281 (53%)	253 (47%)	232 (43%)	302 (57%)	232 (43%)	147 (28%)	155 (29%)	254 (48%)	123 (23%)	157 (29%)	534

^1^As determined by pathologist 1; ^2^Group 1 (≤ 2.0 cm and well or ≤ 1.0 cm and moderate); Group 2 (>2.0 cm and well, or 1.1 to 2.0 cm and moderate, or ≤ 2.0 cm and poor); Group 3 (>2.0 cm and moderate/poor).

### Relative risks for breast cancer death: ER-positive patients

In ER-positive patients treated with tamoxifen, the risk for breast cancer death was positively associated with *HOXB13:IL17BR*, MGI, MGI+*HOXB13:IL17BR*, and BCI in models without tumor size and tumor grade (Table 3). The relative risks (RR) remained elevated for the higher categories of each risk classifier when tumor size and grade were added to the models, although only the RR for high risk defined by the BCI remained statistically significant.

**Table 3:** Relative risks (RR) of breast cancer death associated with each risk classifier among ER-positive patients, stratified by treatment with tamoxifen.

	Cases		Controls					
Risk classifier	n	%	n	%	RR^1^	95% CI	RR^2^	95% CI
*Tamoxifen treated (49 cases and 112 controls)*								
*HOXB13:IL17BR*								
Low risk	19	39%	58	52%	1.0	ref	1.0	ref
High risk	30	61%	54	48%	1.9	0.9 - 3.7	1.5	0.7 - 3.2
MGI								
Low risk	12	24%	55	49%	1.0	ref	1.0	ref
High risk	37	76%	57	51%	2.9	1.3 - 6.2	2.1	0.8 - 5.5
MGI+*HOXB13:IL17BR*								
Low risk	12	24%	55	49%	1.0	ref	1.0	ref
Intermediate risk	14	29%	31	28%	1.8	0.7 - 4.8	1.3	0.4 - 4.2
High risk	23	47%	26	23%	3.5	1.5 - 8.1	2.7	0.9 - 7.5
BCI								
Low risk	13	27%	61	54%	1.0	ref	1.0	ref
Intermediate risk	15	31%	29	26%	2.2	0.9 - 5.6	2.1	0.8 - 6.0
High risk	21	43%	22	20%	4.2	1.7 - 10.3	3.3	1.1 - 10.3
*Tamoxifen untreated (90 cases and 174 controls)*								
*HOXB13:IL17BR*								
Low risk	41	46%	101	58%	1.0	ref	1.0	ref
High risk	49	54%	73	42%	1.6	1.0 - 2.7	1.3	0.8 - 2.3
MGI								
Low risk	32	36%	99	57%	1.0	ref	1.0	ref
High risk	58	64%	75	43%	2.6	1.5 - 4.8	1.7	0.8 - 3.4
MGI+*HOXB13:IL17BR*								
Low risk	32	36%	99	57%	1.0	ref	1.0	ref
Intermediate risk	27	30%	39	22%	2.5	1.3 - 4.8	1.7	0.8 - 3.6
High risk	31	34%	36	21%	2.9	1.4 - 5.8	1.6	0.7 - 3.8
BCI								
Low risk	30	33%	108	62%	1.0	ref	1.0	ref
Intermediate risk	26	29%	28	16%	3.9	1.9 - 8.0	3.2	1.5 - 6.8
High risk	34	38%	38	22%	3.6	1.8 - 7.4	2.0	0.8 - 4.9

^1^Univariate conditional logistic regression models include *HOXB13:IL17BR*, MGI, MGI+*HOXB13:IL17BR *or BCI; ^2^multivariable conditional logistic regression models include *HOXB13:IL17BR*, MGI, MGI+*HOXB13:IL17BR *or BCI plus tumor size (≤2.0,>2.0) and grade (well, intermediate, poor).

Similarly, in ER-positive patients not treated with tamoxifen, the risk for breast cancer death was positively associated with each of the four risk classifiers (Table 3). However, none of the RRs for the high-risk categories remained statistically significant after tumor size and grade were added to the models. Associations were generally weaker in magnitude among ER-positive patients without (versus with) tamoxifen therapy.

Increased risk for breast cancer-specific mortality was associated with higher tumor grade in tamoxifen-treated ER-positive patients and with both higher tumor grade and larger tumor size in tamoxifen-untreated ER-positive patients (data not shown). These associations remained evident, although not necessarily statistically significant, after adding MGI, MGI+*HOXB13:IL17BR *or BCI to the models.

When estimating RRs for breast cancer-specific mortality for different time periods after initial breast cancer diagnosis, results suggested that risk classifiers were better predictors of short (<5 years) vs. long-term (≥5 years) mortality (data not shown). In other sensitivity analyses, RRs generated for ER-positive patients changed little when further adjusting for HER2 status.

### Absolute risk of breast cancer death at 10 years: ER-positive patients

**Figure 1:**
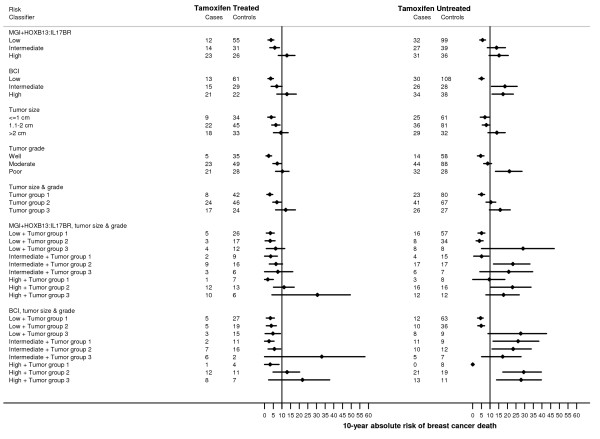
**Estimated 10-year absolute risks of breast cancer death associated with each risk classifier, tumor size, and tumor grade among ER-positive patients, stratified by treatment with tamoxifen**. Each diamond symbol denotes the absolute risk of breast cancer death at 10 years (y-axis). The horizontal line through each diamond symbol denotes the corresponding 95% CI. The vertical lines mark a 10-year absolute risk of 10%, the conventional cut point used in deciding whether or not breast cancer patients should receive adjuvant chemotherapy.

Figure 1 presents the absolute risk of breast cancer death at 10 years post-diagnosis associated with each risk classifier for tamoxifen-treated and tamoxifen-untreated ER-positive patients. The 10-year absolute risks of breast cancer death for ER-positive, tamoxifen-treated patients were 3.7% (95% CI 1.9% to 5.4%), 5.9% (95% CI 3.0% to 8.6%), and 12.9% (95% CI 7.9% to 17.6%) for those in the low-, intermediate- and high-risk groups, respectively, when classified by MGI+*HOXB13:IL17BR*. Risks were similar in magnitude when classified by BCI, with corresponding estimates of 3.5% (95% CI 1.9% to 5.1%), 7.0% (95% CI 3.8% to 10.1%), and 12.9% (95% CI 7.1% to 18.3%). In comparison, those for ER-positive patients not treated with tamoxifen were generally higher, being 5.7% (95% CI 4.0% to 7.4%), 13.8% (95% CI 8.4% to 18.9%), and 15.2% (95% CI 9.4% to 20.5%) when classified by MGI+*HOXB13:IL17BR *and 5.1% (95% CI 3.6% to 6.6%), 18.6% (95% CI 10.8% to 25.7%), and 17.5% (95% CI 11.1% to 23.5%) when classified by BCI.

When classifying ER-positive patients by tumor size and grade alone into three groups (of increasing tumor size and grade), the absolute 10-year risks of breast cancer death were 3.0% (95% CI 1.3% to 4.7%), 7.1% (95% CI 4.7% to 9.5%), and 12.2% (95% CI 6.3% to 17.8%) among those treated and 5.1% (95% CI 3.3% to 6.9%), 10.5% (95% CI 7.7% to 13.3%), and 15.9% (95% CI 9.6% to 21.7%) among those untreated. Although fairly comparable in magnitude to the MGI+*HOXB13:IL17BR*-classified and BCI-classified risk groups (presented above), these estimates varied when further cross-classified by MGI+*HOX13:IL17BR *or BCI.

Of those who died within five years of initial diagnosis, 29%, 30% and 41% were classified by MGI+*HOXB13:IL17BR *and 37%, 24%, and 39% were classified by BCI as low, intermediate and high risk, respectively. Of those who died between five and ten years, 44%, 29% and 27% were classified by MGI+*HOXB13:IL17BR *and 52%, 23%, and 25% were classified by BCI as low, intermediate and high risk, respectively. Of those who died 10 or more years after diagnosis, 53%, 26% and 21% were classified by MGI+*HOXB13:IL17BR *and 58%, 10%, and 32% were classified by BCI as low, intermediate and high risk, respectively.

## Discussion

In a large, independent population of patients with lymph node-negative invasive breast cancer not treated with chemotherapy, we were able to validate reported associations of MGI+*HOX13:IL17BR *and BCI with breast cancer death among ER-positive patients treated with tamoxifen. We also noted associations, although less pronounced for increasing risk categories of MGI+*HOX13:IL17BR *and BCI, among ER-positive patients not treated with tamoxifen. Both risk classifiers appeared to provide risk information beyond tumor size and tumor grade and classified over 50% of ER-positive patients as low risk for breast cancer death.

Several methodological limitations should be considered when interpreting our findings. Since we lacked ER status from the medical records for a substantial proportion of patients, we defined ER status based on gene expression. However, the risk estimates were not materially changed when analyses were restricted to the 84% of patients with ER status documented in the medical records (data not shown). Due to the diagnosis years of the study (1985 to 1994), only 30% of patients received tamoxifen treatment. While this proportion is consistent with what has been reported for other patient populations during this period [15], the number of tamoxifen-treated patients was limited for analysis. Given that the cases and controls were matched with respect to tamoxifen treatment, we could not directly examine whether MGI+*HOXB13:IL17BR *and BCI are able to identify patients who are likely to respond to tamoxifen therapy. We did find a slightly stronger association between each risk classifier and risk for breast cancer death among tamoxifen-treated than tamoxifen-untreated patients, suggesting that both risk classifiers capture response to tamoxifen therapy as well as prognosis. This observation is consistent with evidence that *HOXB13:IL17BR *is associated with response to tamoxifen [3].

In recent years, a number of gene expression signatures have been developed in an attempt to classify the likely prognosis and/or response to therapy for patients with invasive breast cancer better than can currently be done with standard histopathologic features, such as tumor size, tumor grade, ER and HER2 status [16]. Each of these gene expression signatures requires validation in multiple independent populations of patients before its clinical utility can be firmly established. Although only one, MammaPrint, has been FDA approved, there are at least three others that are commercially available, including Oncotype DX, MapQuant Dx, and BCI. MammaPrint and Oncotype DX are currently being examined in prospective clinical trials of breast cancer treatment in both adjuvant and neoadjuvant settings. Many of the others are undergoing validation in retrospective samples from clinical trial populations, in defined cohorts of patients treated in referral centers or the community setting, or in convenience samples of patients from a variety of sources. Both MammaPrint and Oncotype DX are multi-marker signatures (70 and 21 genes, respectively) that include genes from several important carcinogenic pathways (for example, proliferation and hormone response). BCI and MapQuant Dx include a set of genes to specifically provide a genomic signature for tumor grade. There are only a small number of genes, however, that appear in more than one of the many signatures.

In our validation study, the performance of MGI+*HOXB13:IL17BR *and BCI were evaluated using the same risk categories defined in previous studies, specifically among well-characterized subgroups of ER-positive breast cancer patients who did and did not receive tamoxifen. For each patient, pathology reports and diagnostic slides were systematically reviewed to confirm breast cancer diagnosis and uniformly assess tumor size and grade. Course of treatment and vital status were also confirmed by medical record review. In addition, gene expression arrays were conducted blinded to the case-control status of all specimens.

Our results are fairly similar to the three studies that have previously evaluated MGI+*HOXB13:IL17BR *and/or BCI [5,6,17]. We found that the combination of MGI and *HOXB13:IL17BR *signatures was a stronger prognostic indicator than each signature alone among ER-positive breast cancer patients treated with tamoxifen, as demonstrated previously [5], and among those untreated. As in the study by Jerevall *et al. *[6], the RR of breast cancer death was associated with both signatures combined irrespective of whether tamoxifen was received, although the associations that we observed were more pronounced among treated than untreated patients. Analyses limited to post-menopausal breast cancer patients with tumors of ≤ 3.0 cm, the subgroup equivalent to the Jerevall *et al*. study population, yielded associations weaker in magnitude (data not shown). However, our estimated 10-year risks of breast cancer death for tamoxifen-treated and untreated patients classified by MGI+*HOXB13:IL17BR *as low risk (that is, 3.7% and 5.7%) were commensurate to theirs (that is, 2.3% and 5.3%). Risk classification using their BCI algorithm likewise produced comparable results. For tamoxifen-treated and untreated ER-positive patients classified as low risk by the BCI, absolute risks of breast cancer death at 10 years post-diagnosis were 1.1% and 5.1% in their study and 3.5% and 5.1% in ours. In the only other study to evaluate the BCI to date [17], corresponding estimates for low-risk patients receiving adjuvant tamoxifen with or without chemotherapy or adjuvant tamoxifen alone were 3.8% and 7.2%, respectively.

Although both risk classifiers combining MGI and *HOXB13:IL17BR *scores appear to offer added prognostic information, our data suggest that it is still important to consider standard clinicopathologic variables in risk assessment. For example, in analyses that cross-classified ER-positive breast cancer patients by either risk classifier, tumor size, and tumor grade, the 10-year absolute risk of breast cancer death was relatively low for tamoxifen-treated patients presenting with small, low-grade tumors in all three MGI+*HOXB13:IL17BR *or BCI risk groups. This finding, although intuitive, should be interpreted cautiously until confirmed by others, given the limited number of patients studied. It should be further noted that MGI+*HOXB13:IL17BR *and BCI were developed examining ER-positive, lymph node-negative patients treated with tamoxifen. Therefore, the utility of both risk classifiers in determining breast cancer prognosis are not necessarily generalizable to other populations, including patients treated with aromatase inhibitors.

## Conclusions

In an independent population of lymph node-negative invasive breast cancer patients who did not receive adjuvant chemotherapy, we demonstrate that MGI+*HOXB13:IL17BR *and BCI are associated with risk of breast cancer death among ER-positive patients treated and untreated with tamoxifen. Both risk classifiers appeared to capture risk of breast cancer death beyond traditional clinicopathologic indicators and identified a sizable fraction of patients at low risk of mortality. Additional research is needed to determine whether these risk classifiers are also predictors of tamoxifen response and whether their clinical utility for prognostication extends to other subpopulations of breast cancer patients.

## Abbreviations

ACTB: beta-actin; BCI: Breast Cancer Index; BUB1B: budding uninhibited by benzimidazoles 1 homolog beta; CENPA: centromere protein A; CHDH: choline dehydrogenase; Ct: threshold cycle; ER: estrogen receptor; H&E: hematoxylin and eosin; HER2: human epidermal growth factor receptor 2; HMBS: hydromethylbilane synthase; HOXB13: homeobox B13; IHC: immunohistochemistry; IL17BR: interleukin 17 receptor B; IRB: Institutional Review Board; MGI: molecular grade index; NEK2: never in mitosis gene a-related kinase 2; PR: progesterone receptor; RACGAP1: Rac GTPase-activating protein 1; RR: relative risk; RRM2: ribonucleotide reductase M2; RT-PCR: reverse transcription polymerase chain reaction; SDHA: succinate dehydrogenase complex, subunit A; UBC: ubiquitin C.

## Competing interests

The lead author (LAH) and several co-authors (LCS: NSA, DG, LF, and CPQ) are employees of Kaiser Permanente, which received study funds from a contract with bioTheranostics, a bioMerieux company. MGE is currently employed by bioTheranostics and holds company stock options. MGE, X-JM, and DS are patent holders for H:I and MGE is a patent holder for MGI. bioTheranostics did not participate in the decision to submit the manuscript for publication. As part of the funding contract agreement, this was the independent decision of Kaiser Permanente study investigators. However, all authors reviewed and approved the final manuscript. In addition, LAH has received research funding from a grant to Kaiser Permanente from Genomic Health, Inc.

## Authors' contributions

LAH participated in the concept and design of the study, directed the data collection and analysis at Kaiser Permanente and drafted the manuscript. LCS participated in the data analysis, interpretation of the results, and in writing the manuscript. NSA performed statistical analyses and participated in the interpretation of results. X-JM and MGE participated in the concept of the study, development of study methods, data collection and writing the manuscript. DS participated in the development of study methods. DG participated in data collection and in the interpretation of results. LF participated in the interpretation of the results. CPQ participated in the study design, co-directed the data analysis at Kaiser Permanente and participated in the interpretation of results. All authors read and approved the final manuscript.
